# Visuomotor Transformation for the Lead Leg Affects Trail Leg Trajectories During Visually Guided Crossing Over a Virtual Obstacle in Humans

**DOI:** 10.3389/fnins.2020.00357

**Published:** 2020-04-23

**Authors:** Shota Hagio, Motoki Kouzaki

**Affiliations:** Laboratory of Neurophysiology, Graduate School of Human and Environmental Studies, Kyoto University, Kyoto, Japan

**Keywords:** obstacle clearance, visuomotor correction, lower-limb movement, vision, working memory, virtual reality

## Abstract

When walking around a room or outside, we often need to negotiate external physical objects, such as walking up stairs or stepping over an obstacle. In previous studies on obstacle avoidance, lead and trail legs in humans have been considered to be controlled independently on the basis of visual input regarding obstacle properties. However, this perspective has not been sufficient because the influence of visuomotor transformation in the lead leg on the trail leg has not been fully elucidated due to technical limitations in the experimental tasks of stepping over physical obstacles. In this study, we investigated how visuomotor transformation in the lead leg affected movement trajectories in the trail leg using a visually guided task of crossing over a virtual obstacle. Trials for stepping over a physical obstacle were established followed by visually guided tasks in which cursors corresponding to the subject’s lead and trail limb toe positions were displayed on a head-mounted display apparatus. Subjects were instructed to manipulate the cursors so that they precisely crossover a virtual obstacle. In the middle of the trials, the vertical displacement of the cursor only in the lead leg was reduced relative to the actual toe movement during one or two consecutive trials. This visuomotor perturbation resulted in higher elevation not only in the lead limb toe position but also in the trail limb toe trajectories, and then the toe heights returned to the baseline in washout trials, indicating that the visuomotor transformation for obstacle avoidance in the lead leg affects the trail leg trajectory. Taken together, neural resources of limb-specific motor memories for obstacle crossing movements in the lead and trail legs can be shared based on visual input regarding obstacle properties.

## Introduction

Humans can perform locomotion while negotiating external physical objects, such as walking up stairs and stepping over an obstacle. Negotiating obstacles requires an accurate neural representation of the obstacle properties and adaptive spatiotemporal gait modification ability (Drew and Marigold, 2015). Many previous findings provide evidence that visual information about the characteristics and location of the obstacle with respect to the body plays an important role in the planning of gait trajectory modifications (Patla et al., 1996; Mohagheghi et al., 2004; Wilkinson and Sherk, 2005). Individuals fixate on the obstacle for at least two steps before crossing it, which drives transformation of visual information regarding obstacle properties into an appropriate motor command to step over it (Patla and Vickers, 1997).

In obstacle avoidance with both legs, the visual information directly contributes to planning limb elevation in the first leg, or lead leg, whereas continual visual guidance is not needed in the second leg, or trail leg (Patla and Rietdyk, 1993; Patla, 1998; Rhea and Rietdyk, 2007; Lajoie et al., 2012). Previous studies on obstacle avoidance in humans have debated the relationship of limb elevation control between the lead and trail legs. Rhea and Rietdyk (2011) demonstrated that obstacle contact with the trail leg results in changes in toe elevation and clearance in that leg but not in the lead leg in subsequent trials (Rhea and Rietdyk, 2011). The removal of all vision during the last portion close to an obstacle and during obstacle crossing increased the toe elevation height only in the lead leg (Mohagheghi et al., 2004). These previous studies provided the current perspective that sensorimotor transformation based on proprioceptive information regarding the interaction between the obstacle and one limb does not affect the crossing movements in the other leg. Accordingly, lead and trail limb trajectories are considered to be determined based on independent controllers. This knowledge, however, should be revalidated, because the visual information in the affected leg was not available to the other leg movement in the previous studies (Mohagheghi et al., 2004; Rhea and Rietdyk, 2011). The influence of the change of visuomotor transformation in one leg on that in the other leg has not been fully elucidated despite the importance of vision in crossing an obstacle. In this study, we addressed how the modification of sensorimotor transformation in the lead leg based on visual input influences movements in the trail leg.

To date, methods to alter visuomotor transformation in stepping over a physical obstacle have not been proposed. Here, we constructed a new experimental paradigm of obstacle avoidance with visuomotor perturbation using a clearance task over a virtual visual obstacle (Kim et al., 2018). Visuomotor perturbation tasks have been performed in many studies to examine the motor response against the perturbation; for example, a cursor representing the hand position was laterally translated from the current hand position during visually guided reaching movements (Saunders and Knill, 2003; Franklin and Wolpert, 2008; Veyrat-Masson et al., 2010). In the present task, subjects manipulated two cursors representing the lead and trail limb toe positions displayed on the screen of a head-mounted display so that they stepped over a virtual visual obstacle. The virtual obstacle avoidance task makes it possible to experimentally operate the behavior of visually guided toe trajectories in the lead leg and then investigate the effect on the trail limb toe trajectories. Altogether, we could clarify the influence of novel visuomotor transformation in the lead leg on that in the trail leg.

Therefore, the first objective of this study was to construct an experimental paradigm of a virtual obstacle avoidance task and then to verify whether the virtual task could be used to understand motor control in crossing a physical obstacle. Using this method, we then examined how the alteration of visuomotor transformation in the lead leg affected movement trajectories in the trail leg during obstacle crossing in humans. We hypothesized that toe elevation height in the trail leg is corrected with the change of visuomotor transformation in the lead leg without visual information about the trail limb trajectories if visuomotor transformation in the lead leg affected movement in the trail leg. This study will make a significant contribution to understanding the interaction between lead and trail limb motor control in stepping over an obstacle.

## Materials and Methods

### Participants

Thirteen healthy adults (8 males and 5 females, age = 24.3 ± 4.3 years, height = 169.5 ± 8.8 cm, weight = 63.9 ± 14.7 kg, mean ± SD) participated in this study. All subjects had normal/corrected vision and no history of musculoskeletal or neurological disorders. None of the subjects had any knowledge of the purpose of the study, apart from being told that it was aimed at understanding the movement strategies during obstacle clearance. Informed consent was given prior to the experiment. The experimental procedures were conducted in accordance with the Declaration of Helsinki and were approved by the Local Ethics Committee of the Graduate School of Human and Environmental Studies, Kyoto University (19-H-2).

### Task for Stepping Over a Physical Obstacle

**FIGURE 1:**
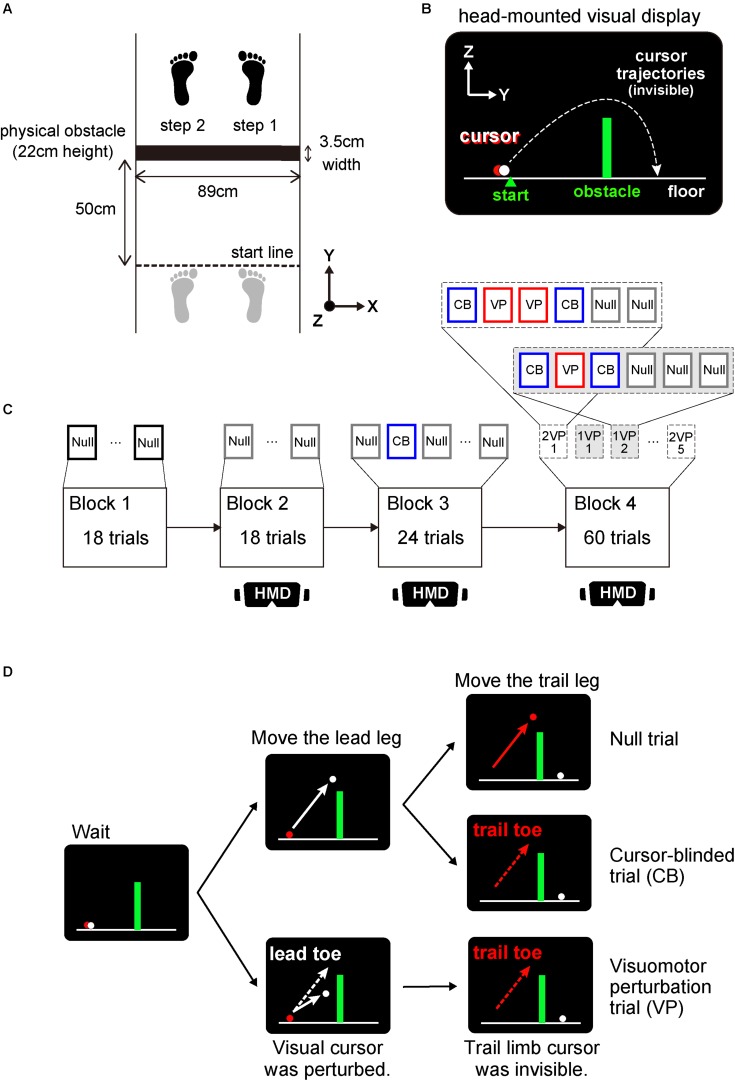
Experimental setup and protocol. **(A)** overhead view of the sequence of foot placements taken by subjects to step over the obstacle (denoted by a solid black rectangle) in the right (lead; step 1) and left (trail; step 2) legs. Before crossing the obstacle, subjects were instructed to stand with their toes on a start line (dashed black line) drawn on the floor. The initial foot position was shown as gray footprints. **(B)** monitor view of a head-mounted display (HMD) apparatus during stepping over a virtual visual obstacle. Two-dimensional coordinates in *Y* and *Z* axes corresponds to the coordinates shown in **(A)**. Subjects manipulated the two cursors representing lead (white) and trail (red) limb toe positions. **(C)** Organization of an experimental session. In the Block 1, 18 physical-obstacle crossing trials were performed followed by the Block 2 with 18 trials that required visually guided stepping over a virtual obstacle. In the Block 3, 8 cursor-blinded (CB) trials in which the cursor representing only the trail limb toe position was blinded were randomly interleaved in every 3rd virtual obstacle crossing trial. The Block 4 contained 10 sets of 6 consecutive trials consisting of one or two visuomotor perturbation (VP) trials sandwiched between pairs of CB trials and followed by either two or three null trials. The illustration labeled HMD indicates that subjects wore an HMD apparatus in the blocks. **(D)** The difference of cursor and toe trajectories in lead and trail legs across each null, CB and VP trial. Both in CB and VP trials, the cursor (red) corresponding to trail limb toe position was invisible.

During the physical tasks, subjects were required to step over an obstacle (Figure 1A). The obstacle was 89 cm wide with a depth of 3.5 cm and a height of 22 cm. The size of obstacle was within the range of that used in the previous studies (width: 57–100 cm; depth: 0.3–10 cm; height: 1–30 cm; Patla and Vickers, 1997; Mohagheghi et al., 2004; Rhea and Rietdyk, 2011; Lajoie et al., 2012; Kim et al., 2018). At the beginning of a trial, subjects were instructed to stand rigidly with their toes precisely on a start line drawn on the floor. The obstacle was placed 50 cm in front of the start line. After stepping over the obstacle with their right leg (i.e., lead leg), subjects paused while straddling the obstacle between the both legs for 2 s. This delay period was set particularly for a virtual task described below, which was shorter than that used in previous studies on working memory regarding obstacle height in humans (more than 5 s; Lajoie et al., 2012; Shinya et al., 2012). Then, subjects cleared the obstacle with their left leg (i.e., trail leg). After a trial, subjects returned to the start line. All movement initiation timings were verbally instructed by an experimenter.

### Experimental Setup for Virtual Visual Obstacle Avoidance Task

Realtime tracking and display of toe positions in MATLAB (R2018a, The MathWorks Inc., Natick, MA, United States) figure enabled us to construct a task for stepping over a virtual visual obstacle. Toe trajectories on each leg were sampled at 100 Hz by the three-dimensional optical motion capture system (OptiTrack V100:R2, Natural Point Inc., Oregon, United States) with 10 cameras spaced around subjects. Three infrared reflective markers were attached on the toe in each leg to create the rigid body. To extract motion capture data from Motive 2.0.2 software (Natural Point Inc., Oregon, United States), the MATLAB Wrapper Class from the NatNet Software Development Kit provided by OptiTrack was used (Maselli et al., 2017). This allowed for rigid body coordinates to be streamed to MATLAB. Several functions were written in MATLAB for finding mechanical quantities based on rigid body coordinates. These functions were then called programmatically by the Java script using the MATLAB Engine API for Java provided by MathWorks.

Just before the tasks in the virtual visual condition, subjects wore a head-mounted display apparatus (PlayStation VR, Sony Interactive Entertainment, Tokyo, Japan), which occluded direct vision of their own bodies and the landscape around them. The headset display was synchronized with a computer screen that captured the toe trajectories. The lead and trail limb toe positions on a sagittal plane were displayed in the 2-dimensional coordinates of a MATLAB figure at 100 Hz as white and red cursors, respectively. The start position was drawn as a triangle on the left side of the screen. The virtual visual obstacle that corresponded to the physical obstacle with a depth of 3.5 cm, a height of 22 cm and placed 50 cm in front of the start position was also displayed. The drawing of the figure and the running of tasks were implemented using custom-made MATLAB script. One experimenter stood behind and slightly to the side of the subjects to prevent a fall.

### Visually Guided Stepping Task Over a Visual Obstacle

Subjects manipulated the two cursors representing the lead and trail limb toe positions displayed on the screen of a head-mounted display apparatus (Figure 1B). At the beginning of each trial, subjects were instructed to set two cursors precisely on a start position. After a 2.5 s holding period, subjects moved the white cursor corresponding to the toe position in their right leg (i.e., lead leg) and cleared the virtual obstacle. The red cursor representing the toe position in the left leg (i.e., trail leg) was then maneuvered to step over the virtual obstacle in 2 s. This delay period was set to encourage attention to the obstacle and the cursor corresponding to the toe position in the trail leg. After 1.5 s, subjects were instructed to move both cursors and return backwards to the start position by sliding their feet. All instructions about holding, movement initiation and going back to the start line were shown in the center of the screen as the messages, “wait,” “go” and “go back home,” respectively. If subjects moved the cursor over 50 cm on the right or left side, a warning message, “attention to the right or left,” was displayed. Subjects were told by an experimenter that a physical object corresponding to the configuration of the virtual object was placed in front of them, although there was indeed no physical object.

### Experimental Procedure in Physical and Virtual Obstacle Tasks

The experiment began with a block of 18 trials of clearing a physical obstacle (Block 1 in Figure 1C). This was followed by two consecutive blocks of 18 and 24 trials, respectively, of stepping over a virtual visual obstacle (Blocks 2 and 3 in Figure 1C). Every 3rd trial in Block 3, a cursor-blinded trial was randomly interleaved (Figure 1D). In the cursor-blinded trial, the cursor representing only the trail limb toe position was blinded throughout the trial. Note that subjects were instructed to step over a virtual obstacle in front of them without visual information about the trail limb toe trajectories. In Block 4, one or two visuomotor perturbation trials were sandwiched between pairs of cursor-blinded trials (Block 4 in Figure 1C). During the visuomotor perturbation trial, the vertical migration length of the cursor corresponding to the lead leg was altered 0.6-fold relative to the actual toe movement (Figure 1D). Consequently, successful clearance of the virtual visual obstacle required elevation of the lead limb toe at least 37 cm. After each consecutive trial, i.e. [cursor-blinded–perturbation (–perturbation)– cursor-blinded], either two or three trials in which both cursors representing the lead and trail limb toe positions were visible were presented so that each set, referred to as “perturbation sets,” consisted of six consecutive trials. Each perturbation set with either one or two perturbations was assayed five times in pseudorandom order. Generally, in the studies using visuomotor perturbation, the perturbation was applied more consistently throughout a lot of trials (Imamizu et al., 1995; Krakauer et al., 2000). In the number of consecutive obstacle clearance trials, however, the toe height gradually decreases potentially due to fatigue or the process to search the optimal strategy (Rhea and Rietdyk, 2011). These factors will make it complex to identify whether the change of toe height is owing to the corrective response against the perturbation or the other factors. To avoid the confusion, we have selected the task where one or two perturbation was applied between the cursor-blinded trials to quantify the spontaneous corrective response to the perturbation (Albert and Shadmehr, 2016).

### Data Collection and Analysis

Three-dimensional lead and trail limb toe positions streamed from Motive software were stored at 100 Hz via custom-written MATLAB (R2018b, Mathworks, Natick, MA, United States) software. The data were high-pass filtered at 5 Hz using a zero-phase-lag 2nd-order Butterworth filter. Maximum toe elevation for the lead and trail legs was defined as the maximum vertical position of each leg’s toe marker during the stepping trajectory over the obstacle. All data were processed using custom-written MATLAB programs.

### Statistics

We calculated Pearson correlation coefficient between the mean vertical heights of toe elevation while stepping over the virtual visual and physical obstacles across each subject. In addition, two-way repeated measures ANOVA was used to test the difference in the height of toe elevation between perturbation sets and trial conditions in each set. Once a significant main effect of condition was observed, *post hoc* tests using Tukey’s method were used to compare the height of toe elevation in the baseline of the perturbation sets with that after visuomotor perturbation. For all the statistical tests, the data points exceeding 3 scaled median absolute deviations away from the median were defined as outliers and were removed. An α threshold of 0.05 was used throughout to assess statistical significance.

## Results

### Association of Motor Performance in Stepping Over Physical and Virtual Obstacles

**FIGURE 2:**
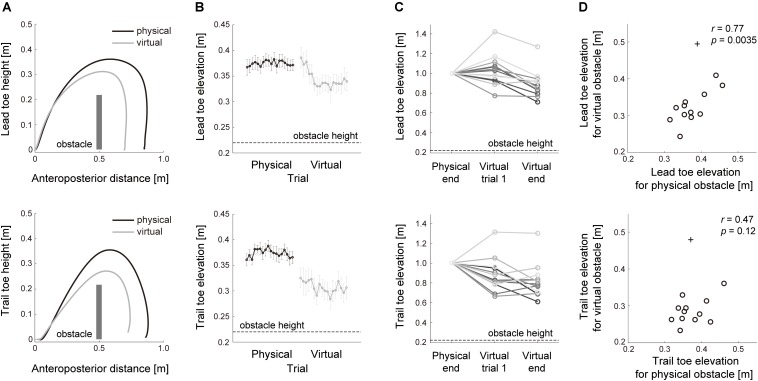
Motor performance in stepping over physical and virtual visual obstacles. **(A)** lead (*top*) and trail (*bottom*) limb toe trajectories while crossing physical (black line) and virtual (gray line) obstacles. The toe trajectories are the average for all subjects from trials 11 to 18 in the Blocks 1 and 2, respectively, that required stepping over the physical and virtual obstacles. The anteroposterior position and height of the obstacle was shown as a dark gray bar relative to the start position. **(B)** The trial-by-trial changes of toe elevation height in 36 consecutive trials in the Blocks 1 (black line) and 2 (gray line). Error bars represent the standard error of the mean. The obstacle height is shown as a black dotted line. **(C)** Toe elevation across each subject in 3 consecutive phases; trials 11–18 in Block 1; the trial at the beginning of Block 2; trials 11–18 in Block 2. The obstacle height is shown as a black dotted line. **(D)** The relationship of toe elevation between physical and virtual obstacles. The circles represent the mean values calculated in each subject, and + signs indicate outliers.

We first verified whether the relationship of the lead leg to the trail leg during clearance of a physical obstacle was examined from the tasks with a virtual obstacle. To this end, the association of motor performance between crossing movements over physical and virtual obstacles was investigated. The vertical toe elevation both in the lead and trail legs decreased when subjects cleared the virtual visual obstacle despite the requirement of the vertical toe elevation to be the same as that of stepping over a physical obstacle (Figure 2A). Just after switching from the physical to the virtual visual tasks, however, the toe elevation for the lead leg was close to that at the end of crossing the physical obstacle (Figures 2B,C), indicating the possibility that a prior history of toe elevation for the physical obstacle remained during crossing movements over the virtual visual obstacle. Association with motor performance was also observed as the common strategy for how high each subject raised his or her feet during obstacle clearance. Across each subject, the vertical heights of lead toe elevation while stepping over the virtual visual obstacle were strongly correlated with those while clearing the physical obstacle (Figure 2D, lead leg; *r* = 0.77, *p* = 0.0035). This interaction of motor performance between the different environments indicates that the task with a virtual visual obstacle can examine the control strategies in stepping over the physical obstacle. In the case of the trail leg, however, the relationship of the toe height between physical and virtual environments was lower as compared with that in the lead leg (Figure 2D, trail leg; *r* = 0.47, *p* = 0.12).

### Adaptable Change of Lead Toe Elevation During Obstacle Clearance With Visuomotor Perturbation

**FIGURE 3:**
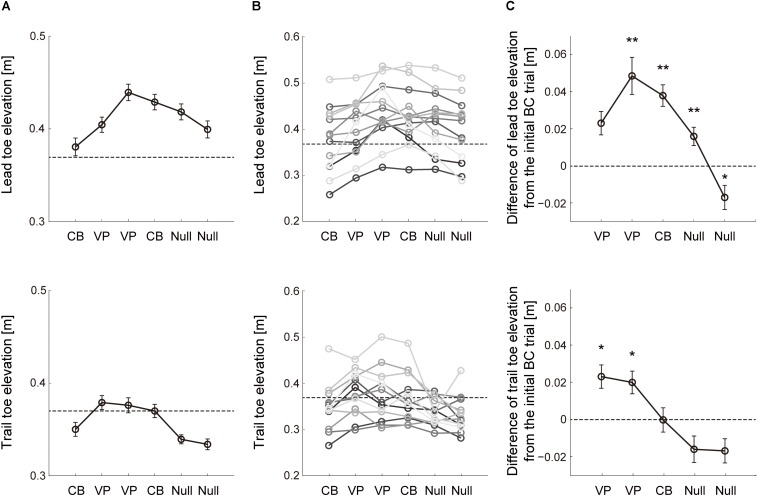
Change of toe elevation due to 2 consecutive visuomotor perturbations. **(A)** Lead (*top*) and trail (*bottom*) limb toe elevations during sets of 6 successive trials with two visuomotor perturbation (VP) trials in Block 4 (Figure 1C). The data are the mean values for all repetitions and subjects. Error bars represent the standard error of the mean. Dashed line represents the lowest value of the desired toe height during the perturbation. **(B)** Mean toe heights in Block 4 across each subject. Dashed line represents the lowest value of the desired toe height during the perturbation. **(C)** Difference of the toe height between each trial and the initial cursor-blinded trial on each perturbation set. The data are the mean values for all repetitions and subjects. Error bars represent the standard error of the mean. Asterisk means statistically significant difference in the toe heights from the initial trial to each following trial; *p < 0.05 and **p < 0.001 using the *post hoc* Tukey test.

The vision-based toe trajectory modification during obstacle avoidance was examined with repeated perturbation sets composed of six consecutive trials in Block 4 (Figure 1C). The mean height of the lead limb toe elevation for all subjects was shown in the six trials with two consecutive perturbations (Figure 3A, lead leg). Once the visuomotor perturbation was applied in the second trial, toe elevation increased compared with the first trial. The difference in toe height between the first and second trials reflected feedback correction for the visuomotor perturbation during movement, whereas the change in toe height that occurred from the first trial to each of the other trials reflected both within-movement feedback correction and predicted movement after offline correction. Modification of lead limb toe elevation was consistently observed in individual subjects (Figure 3B, lead leg). Two out of 13 subjects, however, did not modify the toe elevation that reached into the required height, i.e., 37 cm, after visuomotor perturbation. These subjects needed to increase their toe elevation on the perturbation trial compared to others because the toe height in the first baseline trial was lower than the other subjects.

Lead limb toe height was quantified relative to the first trial on each perturbation set across each subject (Figure 3C, lead leg). It can be clearly seen that subjects effectively scaled lead leg elevation in response to the perturbation trials (*F*_5,59_ = 11.81, *p* = 5.7 × 10^–8^). *Post hoc* tests indicated that the toe heights in the lead leg were higher than those in the first trial during the two consecutive visual perturbations (*p* = 0.059 and 0.0045). In the following washout trials, i.e., from the fourth to sixth trials, the lead toe height was returning to the baseline value (*p* = 0.0042, 3.22 × 10^–4^ and 0.049). These results indicate that the vision-based motor plan for stepping over the obstacle with the lead leg was modified using the virtual visual obstacle avoidance task.

### Effect of Visual-Based Motor Planning in the Lead Leg on the Trail Leg While Stepping Over an Obstacle

As in the lead leg, the trail limb toe elevation was quantified on each perturbation set with two consecutive visuomotor perturbation trials (Figure 3A, trail leg). Note that the cursor representing the trail limb toe position was invisible in both cursor-blinded and perturbation trials (Figure 1D). Hence, the alteration in toe height observed from the second to the fourth trials depended on the movement in the lead leg. Before and after the cursor in the lead leg was perturbed, significant effect was observed in the trail limb toe elevation (*F*_5,59_ = 8.18, *p* = 6.15 × 10^–6^). *Post hoc* tests indicated that the trail limb toe trajectories in the second and third trials were elevated higher relative to the baseline despite the lack of visual information regarding the trail leg (Figure 3C, trail leg; *p* = 0.024 and 0.013), indicating that the trail limb toe trajectories were modified based on the vision-based errors in the lead leg. In several subjects, trail limb toe elevation was not comparable to the required height after the visuomotor perturbation (Figure 3B, trail leg). According to the result, the effect of the visuomotor error in the lead leg on the trail limb movement was smaller than that on the lead leg itself. The trail limb toe height was then modified toward the baseline value from the fourth to sixth washout trials where the perturbations were removed (*p* = 0.059, 0.94, and 0.28).

**FIGURE 4:**
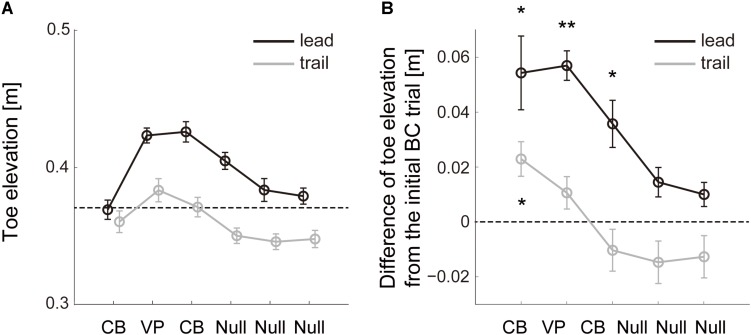
Change of toe elevation due to one visuomotor perturbation. **(A)** Lead (black line) and trail (gray line) limb toe elevation during sets of 6 successive trials with one visuomotor perturbation (VP) trial in Block 4 (Figure 1C). The data are the mean values for all repetitions and subjects. Error bars represent the standard error of the mean. Dashed line represents the lowest value of the desired toe height during the perturbation. **(B)** Difference of the toe height between each trial and the initial cursor-blinded trial on each perturbation set. The data are the mean values for all repetitions and subjects. Error bars represent the standard error of the mean. Asterisk means statistically significant difference in the toe heights from the initial trial to each following trial; *p < 0.05 and **p < 0.001 using the *post hoc* Tukey test.

The modification of trail limb toe trajectories on the basis of the visual errors in the lead leg was also observed in the perturbation sets with one visuomotor perturbation trial (Figure 4). There was significant effect due to the perturbation both in the lead and trail limb toe elevation (*F*_5,59_ = 13.13, *p* = 1.22 × 10^–8^; *F*_5,59_ = 8.18, *p* = 6.15 × 10^–6^). The mean trail limb toe height increased in the second trial without visual input about that toe position (Figure 4B, trail leg; *post hoc* Tukey test, *p* = 0.031) while the lead limb toe height was corrected to be higher from the second to fourth trials (Figure 4B, lead leg; *post hoc* Tukey test, *p* = 0.016, 2.28 × 10^–6^ and 0.013), and then went back to the baseline in the following trials (*post hoc* Tukey test, *p* = 0.15 and 0.28).

## Discussion

The main aim of the present study was to examine the effects of visuomotor transformation in the lead leg on movement trajectories in the trail leg during obstacle crossing in humans. To this end, the experimental paradigm of a virtual obstacle avoidance task was first constructed that makes it possible to alter the visuomotor transformation involved in obstacle crossing. The interactive motor performance between the physical and virtual visual tasks indicated that the virtual visual obstacle task enabled us to examine motor control in stepping over an external physical obstacle. With this available method, we then demonstrated that the trail limb toe trajectories were modified after visuomotor perturbation in visually guided lead limb movement. Therefore, the results suggest that visuomotor transformation in the lead leg contribute to a motor plan for trail limb toe trajectories during obstacle crossing.

According to previous studies on obstacle avoidance in humans, lead and trail legs were considered to be controlled independently on the basis of visual input regarding obstacle properties (Patla and Rietdyk, 1993; Patla, 1998; Rhea and Rietdyk, 2007), and lead leg non-visual sensorimotor signals, proprioceptive information, or efferent copy signals play a relatively minor role in guiding the trail leg trajectory (Lajoie et al., 2012). In contrast, in the present study, the visuomotor error that occurred in visually guided lead limb movement led to correction of the toe elevation height not only in the perturbed lead limb but also in the unperturbed trail limb; therefore, the trail limb movement depended on the sensory error feedback from the lead limb. The bilateral movement correction elicited in response to unilateral perturbations occurred when the task goal was shared between the right and left arms, indicating that sensory feedback from one limb can modify the movement of another limb in a task-dependent manner (Mutha and Sainburg, 2009; Omrani et al., 2013). Although the lead and trail limb toe positions were independently controlled in the present study, the underlying goals of the obstacle clearance task would be shared in both legs. Furthermore, while this study measured the corrective response of the lead and trail limb movements after the one or two visuomotor perturbation trials, the response remained in the following null trials (Figures 3, 4), indicating the learning response against the transient perturbation (Albert and Shadmehr, 2016). In this sense, the bilateral movement correction might reflect interlimb transfer of the visuomotor learning. A previous study on locomotor adaptation demonstrated the interlimb transfer of learning effects on a new obstacle avoidance task occurred when the lead leg became the trail leg, and vice versa (van Hedel et al., 2002). The interlimb transfer was also observed following adaptation to a novel visuomotor condition in visually guided reaching movement (Imamizu and Shimojo, 1995; Sainburg and Wang, 2002). Thus, movement information learned with one limb transfers to the same movements made with the other limb in a task-dependent manner. Despite the interlimb transfer of movements, each limb can also adapt to visuomotor rotation oppositely directed for the two arms (Wang and Sainburg, 2003). The adaptations to opposite visuomotor rotations are known to interfere with each other within the same arm (Krakauer et al., 2000; Tong et al., 2002). The movement information obtained during the opposite arm training is obligatorily competed with subsequent performance with the other arm (Kumar et al., 2018), whereas the limb-specific memories for both arms can be stored (Wang and Sainburg, 2003). Together, these findings suggest that learning of a visuomotor rotation is represented in shared neural resources for the acquisition of motor memories across different limb’s controller. In the case of an obstacle crossing movement, it has been reported that the obstacle properties would be stored in the working memory represented as spatiotemporal neural activity in area 5 of the posterior parietal cortex (Lajoie et al., 2010; Wong and Lomber, 2019). Limb-specific memories might be stored for the lead and trail legs but can be affected by the sensorimotor information in the other limb. Indeed, proprioceptive feedback and an efferent copy signal provided when stepping over an obstacle with the lead limb enhanced memory of the obstacle height that was recalled in the trail limb movement compared with the case in which only visual information was available (McVea and Pearson, 2007; McVea et al., 2009; Shinya et al., 2012). The present study suggested that neural resources of limb-specific motor memories for obstacle crossing movements in lead and trail legs were shared based on visual input regarding the interaction between obstacle properties and limb movements. By contrast, there is the possibility that different explicit strategies were used for control of lead and trail legs (Taylor and Ivry, 2011). Future experiments are needed to examine whether the corrective response in a trail leg after visuomotor correction in a lead leg reflects implicit or limb-specific explicit control for stepping over an obstacle.

Motor skill transfer between physical and virtual visual environments was demonstrated in previous studies that tried to enhance motor performance in the real world based on virtual reality training for sports and rehabilitation (Todorov et al., 1997; Sveistrup, 2004; Adamovich et al., 2009). The virtual environments can present combinations of multimodal stimuli that are not found in the natural world and produce changes in the environment that would not be possible physically. Clinical and rehabilitation therapists or trainers gain unique benefits from being able to control stimuli in virtual reality environments. The virtual reality environment is increasingly used not only for application but also for neuroscience research (Tarr and Warren, 2002; Bohil et al., 2011). Motor tasks guided by visual cues corresponding to actual movements are virtual reality tasks in the broad sense (Krakauer et al., 2000). The present study expanded a visually guided motor task into an obstacle crossing movement and then demonstrated the transfer of motor performance; toe elevation early in the task of crossing over a virtual obstacle was biased by the preceding physical task. The result suggested that the visual perception and the sensorimotor processes engaged in each of the physical and virtual tasks are related to each other. Furthermore, there was a correlation between toe elevation in the physical and virtual tasks, indicating that the common strategies of movement planning to implement successful obstacle crossing were used in these two environments. Therefore, the visually guided task of crossing over a virtual obstacle is an effective experimental paradigm to investigate motor control of coordinated movements in the lead and trail legs during obstacle avoidance, which can take the place of physical tasks. This paradigm has the potential to expand the present experimental setup into motor tasks in novel visuomotor environments with various visual gains or combined with multimodal sensory stimuli. However, the fact remains that there was an apparent gap in the environment and resultant motor performance between the virtual and the physical tasks (Lin et al., 2015). For example, attributes of the obstacle as well as the toe position are available to the trail leg during the null trials of the virtual task whereas these were out of sight in the physical task. The visual information about the obstacle and the toe position only on the sagittal plane was also specific to the virtual task. Whether and how the present results in the virtual task were transferred to the physical environment should be tested in future studies.

In summary, visuomotor perturbation applied only to the lead leg movement in the middle of tasks of crossing over a virtual visual obstacle resulted in trajectory modification not only in the lead leg but also in the trail limb toe, indicating that the visuomotor transformation for obstacle avoidance in the lead leg affects trail leg trajectories. To date, lead and trail legs in humans have been considered to be controlled independently, whereas these results suggest that neural resources of limb-specific motor memories for obstacle crossing movements in lead and trail legs were shared based on visual input regarding obstacle properties and limb trajectories during crossing. The obstacle clearance task in the virtual visual environment is a practical experimental paradigm that makes it possible to flexibly alter spatiotemporal coordination in the visuomotor system regarding obstacle perception and lower-limb movements.

## Data Availability Statement

The raw data supporting the conclusions of this article will be made available by the authors, without undue reservation, to any qualified researcher.

## Ethics Statement

The studies involving human participants were reviewed and approved by the Local Ethics Committee of the Graduate School of Human and Environmental Studies, Kyoto University (19-H-2). The patients/participants provided their written informed consent to participate in this study.

## Author Contributions

SH and MK conceived and designed the experiments, collected the data, drafted the manuscript and revised it critically for important intellectual content, and approved the final version to be published. SH analyzed and interpreted the data.

## Conflict of Interest

The authors declare that the research was conducted in the absence of any commercial or financial relationships that could be construed as a potential conflict of interest.
